# KRT18 regulates trophoblast cell migration and invasion which are essential for embryo implantation

**DOI:** 10.1186/s12958-023-01129-y

**Published:** 2023-08-24

**Authors:** Xiaoling Liang, Xiaoxiao Qiu, Yana Ma, Wenzhi Xu, Sijia Chen, Peipei Zhang, Mengying Liu, Xiaona Lin

**Affiliations:** 1 Assisted Reproduction Unit, Department of Obstetrics and Gynaecology, Sir Run Run Shaw Hospital, Zhejiang University School of Medicine, Hangzhou, China; 2 Assisted Reproduction Center, Northwest Women’s and Children’s Hospital, Xi’an, China; 3 Department of Obstetrics and Gynaecology, Taizhou Municipal Hospital, Taizhou, China; 4 Key Laboratory of Reproductive Dysfunction Management of Zhejiang Province, Hangzhou, China; 5 Department of Obstetrics and Gynaecology, Tiantai People’s Hospital of Zhejiang Province, Taizhou, China

**Keywords:** KRT18, Embryo implantation, Embryo adhesion, E-cadherin, Cell migration, Cell invasion

## Abstract

Female infertility is a worldwide concern that impacts the quality of life and well-being of affected couples. Failure of embryo implantation is a major cause of early pregnancy loss and is precisely regulated by a programmed molecular mechanism. Recent studies have shown that proper trophoblast adhesion and invasion are essential for embryo implantation. However, the potential regulatory mechanism involved in trophoblast adhesion and invasion has yet to be fully elucidated. KRT18 has been reported to play a critical role in early embryonic development, but its physiological function in embryo implantation remains unclear. In the present study, we revealed that KRT18 was highly expressed in trophoblast cells and that knockdown of KRT18 in mouse embryos inhibited embryo adhesion and implantation. In vitro experiments further showed that silencing KRT18 disturbed trophoblast migration and invasion. More importantly, we provide evidence that KRT18 directly binds to and stabilizes cell surface E-cadherin in trophoblast cells through microscale thermophoresis (MST) analysis and molecular biology experiments. In brief, our data reveal that KRT18, which is highly expressed in trophoblast cells, plays an important role in the regulation of trophoblast invasion and adhesion during embryo implantation by directly binding to E-cadherin.

## Introduction

Female infertility affects more than 48 million women worldwide according to an investigation by the World Health Organization and is a major challenge in reproductive medicine [1]. At present, approximately 2% of pregnancies are conceived by using assisted reproductive technology (ART) [2], but the overall success rate of ART remains low [3]. Among numerous factors that cause female infertility, embryo implantation failure is a major cause of early pregnancy loss; however, the molecular mechanisms underlying these processes are not yet fully understood [4]. Therefore, revealing the biophysical mechanism underlying the biological processes of embryo implantation is very important.

The cytoskeleton, which is made up of actin microfilaments, intermediate filaments (IFs), and microtubules, is essential for most cellular functions, such as supporting cell shape and cell adhesion [5]. IFs, whose main protein components are encoded by a large family of differentially expressed genes, are essential for maintaining the structural integrity of tissues [5]. As a type I cytokeratin, keratin 18 (KRT18) is a member of the IF family, which is a very important part of the cytoskeleton [6]. This protein is involved in many cellular processes, including withstanding external stress and maintaining the structural integrity of the cytoplasm and mitochondria [7]. Originally, it was discovered that KRT18 was expressed in endothelial and epithelial cells from the gastrointestinal and respiratory tracts [8]. Subsequently, KRT18 was found to be abnormally expressed in various malignant tumours and was gradually regarded as a diagnostic and prognostic marker in cancers, including gastric cancer [9, 10], non–small cell lung cancer [11], hepatocellular cancer [12], and colorectal cancer [13]. Recently, keratin 8 and 18 (K8/18) were reported to play a critical role during the early development of embryos [14], but their roles in embryo implantation remain unclear.

E-cadherin is known as a cell adhesion molecule. Its primary function is adhesion between epithelial cells and maintenance of the normal epithelial architecture [15]. Results of animal studies have suggested that E-cadherin also plays crucial roles in endometrial receptivity [16]. Disruption of E-cadherin in mouse embryos impaired preimplantation development and implantation [17]. In humans, E-cadherin is believed to be involved in embryo attachment and implantation [18]. E-cadherin expression was found to be significantly reduced in a recurrent miscarriage group compared with a control group [19].

In the present study, we found that KRT18 is highly expressed in trophoblastic blastocyst cells and is localized in the cytoplasm. Knockdown of KRT18 in mouse embryos inhibited embryo adhesion. In vitro experiments showed that silencing KRT18 inhibited trophoblast cell migration and invasion and inhibited JEG-3 sphere adhesion. MST analysis of the His-E-cadherin fusion protein and GST-KRT18 fusion protein showed that KRT18 directly binds to E-cadherin. Knockdown of KRT18 in trophoblastic cells impaired the stabilization of cell surface E-cadherin. These results demonstrated that KRT18 plays a significant role in embryo implantation.

## Results

### The expression pattern and subcellular location of KRT18 in preimplantation mouse embryos

**Fig. 1:**
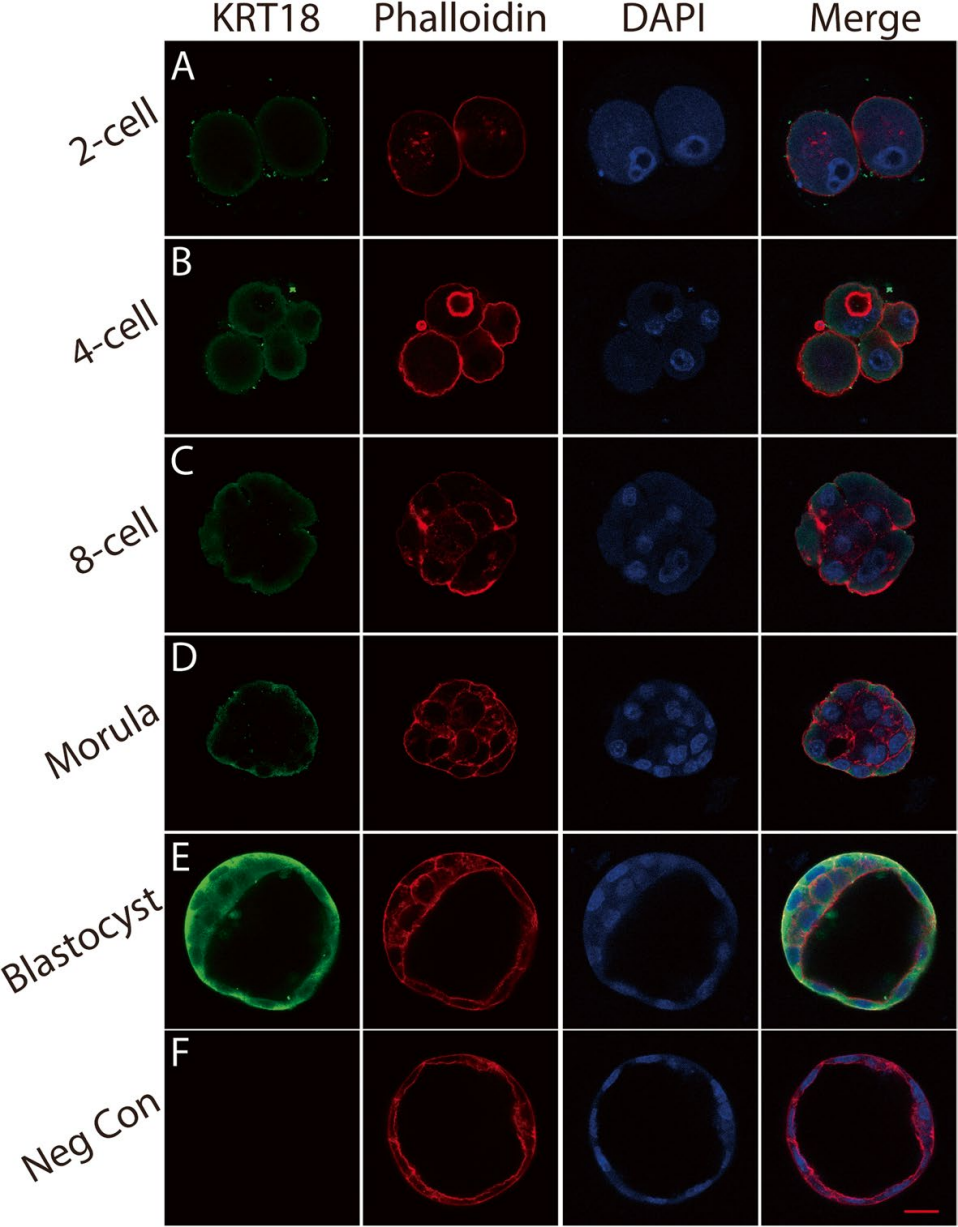
The expression and subcellular localization of KRT18 in preimplantation embryos. **A**-**F** Embryos at the 2-cell, 4-cell, 8-cell, morula and blastocyst stages were harvested for immunofluorescence staining. Anti-KRT18 antibody (green), phalloidin (red) and Hoechst (blue) were used to determine the expression of the corresponding proteins. **A**-**E** The expression level of KRT18 gradually increased during embryo development. KRT18 signalling was clearly observed in blastocysts trophoblasts. **F** A negative control experiment was performed by replacing the anti-KRT18 antibody with IgG. Scale bar, 20 µm

To investigate the expression pattern of KRT18 in preimplantation mouse embryos, we performed immunofluorescence analysis at different stages. As shown in Fig. 1A-F, KRT18 was expressed in trophoblasts at the blastocyst stage before implantation and localized in the cytoplasm (Fig. 1E), while no apparent KRT18 expression was detected during earlier stages (Fig. 1A-D). The F-actin architecture was visualized by phalloidin staining. These findings suggest that KRT18 is expressed in blastocyst trophectoderm cells, which indicates a potential function of KRT18 in the process of embryo implantation.

### Knockdown of KRT18 impairs mouse embryo adhesion and implantation

**Fig. 2:**
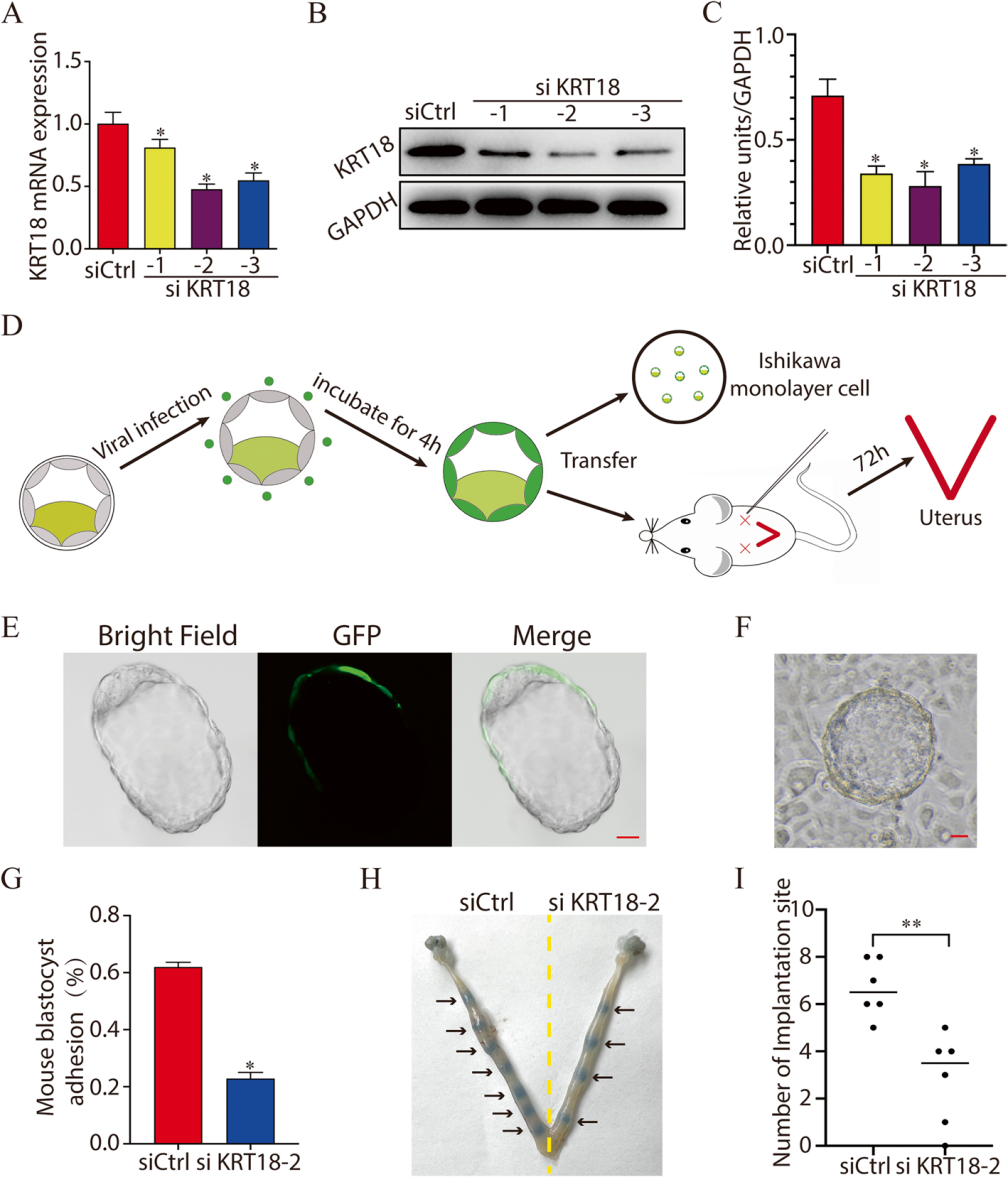
Knockdown of KRT18 impairs mouse embryo adhesion and implantation. **A**-**C** Knockdown of KRT18 with lentivirus reduced KRT18 expression at the mRNA (**A**) and protein levels (**B**) and (**C**). The histogram shows the quantitative analysis of western blotting (**C**). **D** Schematics of the experimental mouse embryo adhesion and in vivo implantation process. **E** Representative picture of embryos treated with lentivirus for 48 h. Scale bar, 20 µm. **F** Representative picture of embryos that adhered to a monolayer of Ishikawa cells. Scale bar, 50 µm. **G** The attachment rate of the mouse embryos was plotted as a histogram. *, *p* < 0.05. Blastocysts treated with control or siKRT18-2 lentivirus were transferred into pseudopregnant mice. Seventy-two hours after transfer, the number of implantation sites in the uteri was counted. (H) Representative picture of implantation sites in the uteri. **I** The number of implantation sites in the uteri with transferred embryos treated with control or siKRT18-2 lentivirus (*n* = 6). The data are presented as the mean ± SE. **P < 0.01

As KRT18 was expressed in trophoblasts at the blastocyst stage (Fig. 1), we hypothesized that KRT18 may play a role in embryo implantation. To test this hypothesis, we employed an embryo trophoblast-specific knockdown model [20]. Knockdown of KRT18 with lentivirus markedly reduced KRT18 mRNA and protein expression in NIH/3T3 cells (Fig. 2A-C). As LV-siKRT18-2 showed the highest interference efficiency among these variants, this fragment was used in the subsequent interference experiments. Figure 2D is a schematic of the experimental process. Figure 2E shows a representative picture of the embryos treated with lentivirus for 48 h in which GFP was detected only in trophoblast cells. Then, mouse embryo adhesion assays were performed. As shown in Fig. 2F and G, the mouse embryo adhesion rate was significantly reduced in the LV-siKRT18-2 group (23.17%) compared with the control group (61.41%). For in vivo experiments, as shown in Fig. 2H and I, the implantation sites were significantly reduced in the LV-siKRT18-2 group compared with the control group (*P* < 0.01). The above results indicated that knockdown of KRT18 in trophoblasts may impair mouse embryo adhesion and implantation.

### Knockdown of KRT18 inhibits trophoblast cell migration and invasion

**Fig. 3:**
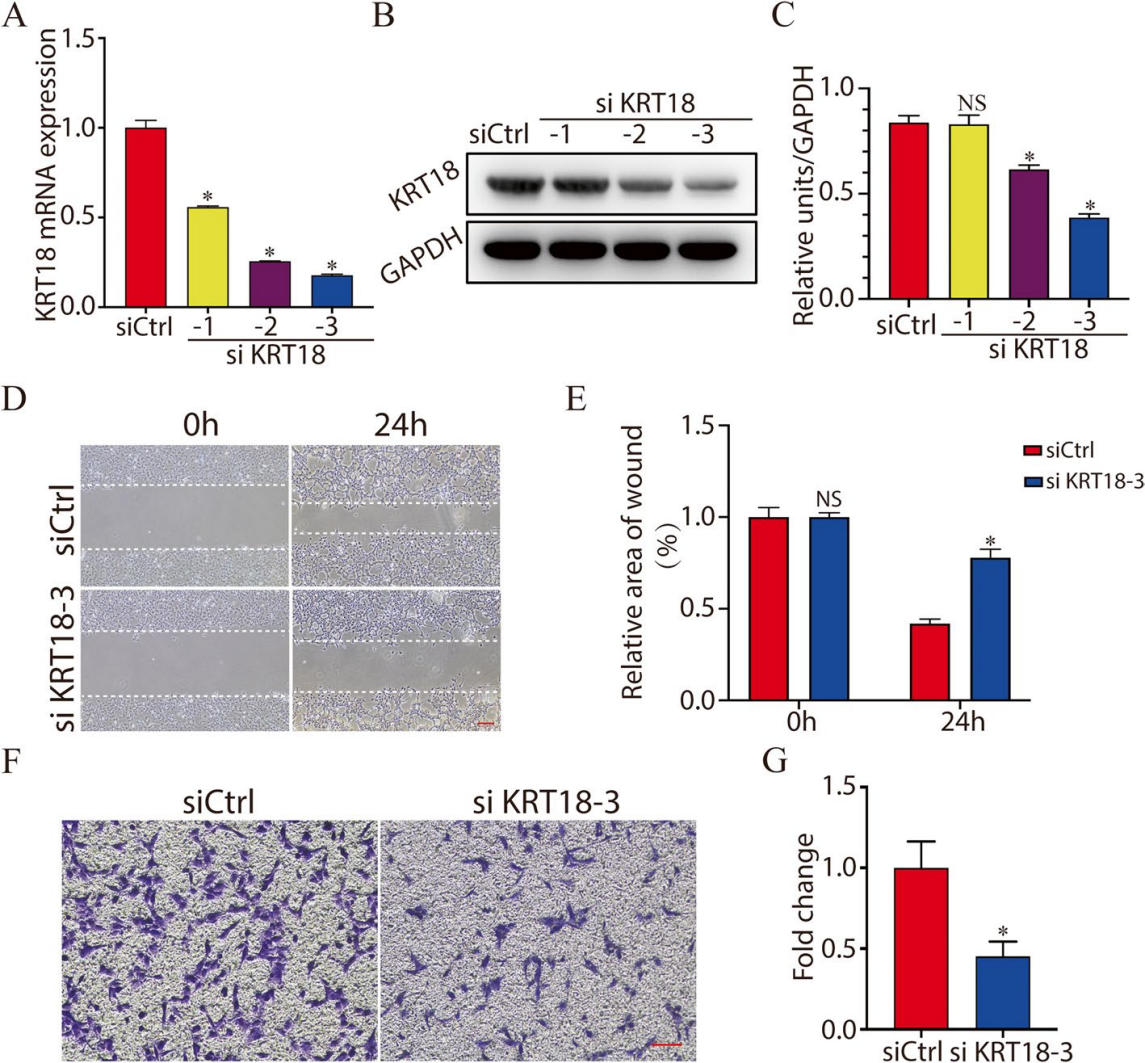
Knockdown of KRT18 inhibits trophoblast cell migration and invasion. **A** and **B** Knockdown of KRT18 with siRNAs reduced KRT18 mRNA (**A**) and protein (**B**) (**C**) expression. **D** and **E** Twenty-four hours after siKRT18-3 transfection, three scratches were made onto a monolayer of HTR8/SVneo cells, and images of the initial scratch and the scratch after 24 h were taken (**D**). Scale bar, 200 µm. The histogram shows the statistical results of the cell scratch assay (**E**). **F** and **G** HTR8/SVneo cell invasiveness was determined using Matrigel transwell assays after 24 h of siKRT18-3 transfection (**F**), and the histogram shows the statistical results (**G**). Scale bar, 100 µm. *, *p* < 0.05

Embryo implantation is a developmental milestone in which the embryo undergoes major reorganization [21]. Cell motility and invasion play crucial roles in embryo implantation. To investigate the function of KRT18 in the process of embryo implantation, we used small interfering RNA (siRNA) to investigate the effect of KRT18 knockdown on the migration and invasion of trophoblast cells. Knockdown of KRT18 with siRNAs markedly reduced KRT18 mRNA and protein expression (Fig. 3A-C). However, siKRT18-3 showed the highest interference efficiency among these variants, and thus, this fragment was used in the subsequent interference experiments. To study whether KRT18 knockdown affects the migration and invasion of HTR8/SVneo cells, we performed a cell scratch assay and transwell invasion assay after siRNA transfection. As shown in Fig. 3D and E, cell migration was significantly inhibited by knockdown of KRT18. Similarly, KRT18 siRNA knockdown significantly reduced cell invasion, as shown by the results of the transwell invasion assay (Fig. 3F and G). The above results indicated that downregulation of KRT18 inhibits trophoblast migration and invasion.

### Knockdown of KRT18 impairs JEG-3 spheroid adhesion

**Fig. 4:**
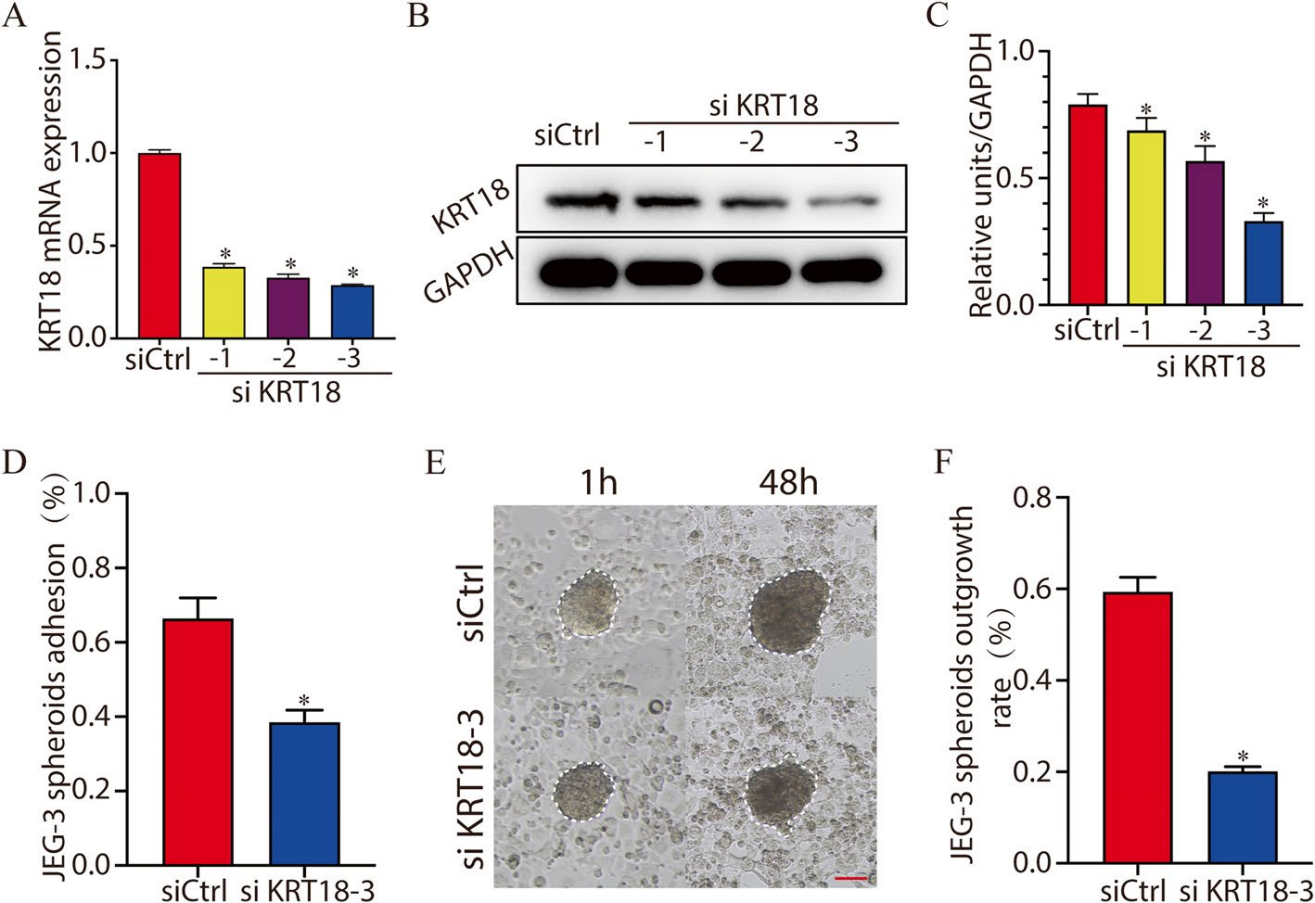
Knockdown of KRT18 impairs JEG-3 spheroid adhesion and outgrowth. **A**-**C** Knockdown of KRT18 with siRNAs reduced KRT18 expression at the mRNA (**A**) and protein levels (**B**). The histogram shows the quantitative analysis of western blotting (**C**). **D** After treatment with siKRT18-3 for 24 h, JEG-3 spheroids were generated and transferred onto a monolayer of Ishikawa cells. After centrifugation of the plate at 145 rpm for 10 min and removal of the spheroids that did not bind, the attachment rate was determined (the number of attached spheroids divided by the total number of input spheroids). The JEG-3 spheroid adhesion rates were plotted as a histogram. **E** and **F** The outgrowth ability of JEG-3 spheroids. The spheroids that attached to the Ishikawa cell monolayer were photographed at 1 h and 48 h after being transferred onto the Ishikawa cell monolayer (**E**). Scale bar, 100 µm. The histogram shows the outgrowth rate of the two groups (**F**). *, *p* < 0.05

For both ethical and technical reasons, human embryos are difficult to obtain. As a human placental choriocarcinoma cell line, JEG-3 has been widely used as an in vitro model for the placenta [22]. Knockdown of KRT18 with siRNAs markedly reduced KRT18 mRNA and protein expression (Fig. 4A-C). However, siKRT18-3 showed the highest interference efficiency among these variants, and thus, this fragment was used in the subsequent interference experiments. To further explore the function of KRT18, we conducted JEG-3 spheroid attachment and outgrowth assays. After treatment with siKRT18-3, JEG-3 spheroids were generated by shaking cells (at a density of 3 × 10^5^ cells per well of 6-well plate) at 88 rpm for 16–18 h under standard cell culture conditions, and then, the spheroids were transferred onto Ishikawa monolayers. After 1 h of coculture, the spheroid attachment rate of the siKRT18-3 group (38.4 ± 2.7%) was significantly decreased compared with that of the control group (66.4 ± 4.5%) (Fig. 4D). Culture was continued for 48 h, and the outgrowth area was measured. As shown in Fig. 4E and F, the outgrowth area of the siKRT18-3 group was significantly reduced. The above results demonstrated that the knockdown of KRT18 impairs JEG-3 spheroid adhesion and outgrowth.

### Knockdown of KRT18 decreased E-cadherin expression

**Fig. 5:**
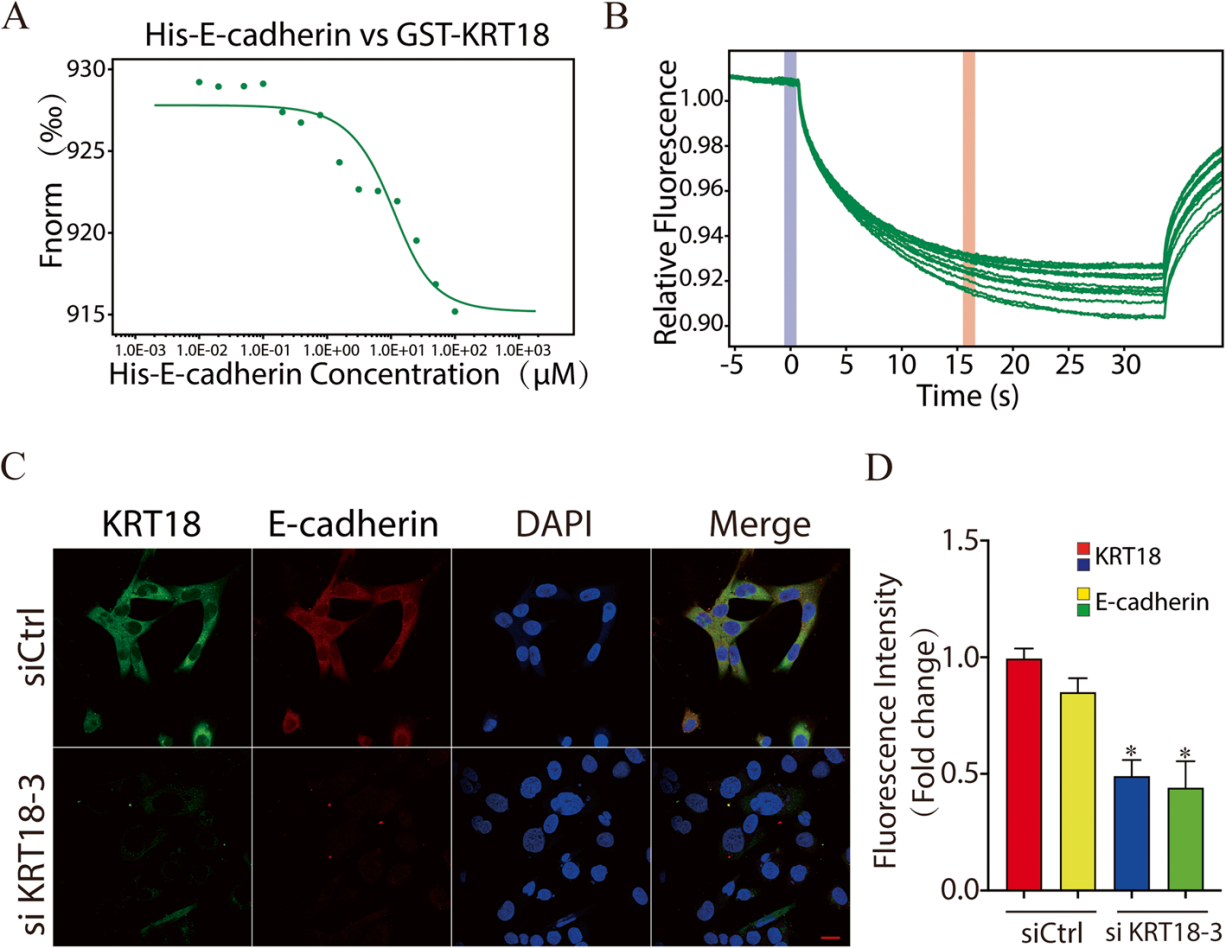
Knockdown of KRT18 impairs the expression of E-cadherin. **A** and **B** MST results of His-E-cadherin fusion protein vs. GST-KRT18 fusion protein. The fluorescence change upon switching the laser on and off at 20% intensity is shown. **C** HTR8/SVneo cells were treated with the control or siKRT18 siRNA. After incubation for 48 h, the cells were harvested for immunofluorescence staining and then photographed with confocal laser scanning microscopy. Anti-KRT18 antibody (green), anti-E-cadherin antibody (red), and Hoechst (blue) were used to determine the expression of the corresponding proteins. The results shown are representative of 3 independent experiments. Scale bar, 20 µm. **D** Quantification of mean fluorescence intensity. KRT18 and E-cadherin fluorescence was normalized with the mean fluorescence intensity of the control group. *P < 0.05

To further elucidate the role of KRT18 in embryo implantation, we used additional in vitro models. Guo et al. [23] employed proximity biotinylation and quantitative proteomics to isolate and identify 612 proteins in the vicinity of E-cadherin’s cytoplasmic tail, which indicated that KRT18 may interact with E-cadherin. To investigate the interplay between these two proteins, we next used microscale thermophoresis (MST). As expected, KRT18 directly bound E-cadherin, with a Kd of 5.5979 μM (Fig. 5A and B). We next investigated whether KRT18 dysfunction affects the expression of E-cadherin. HTR8/SVneo cells were treated with siKRT18-3 siRNA. After 48 h, HTR8/SVneo cell staining revealed that KRT18 siRNA caused a significant decrease in E-cadherin expression (Fig. 5C, D). The above results indicated that knockdown of KRT18 may impair its reaction with E-cadherin and then decrease cell mobility.

## Discussion

Early pregnancy loss is a very common phenomenon in humans, and approximately 75% of pregnancy failures are due to implantation defects [24, 25]. However, repeated implantation failure (RIF) is a major issue. RIF is defined as the inability to achieve a clinical pregnancy in three or more consecutive IVF cycles [26]. In fact, both embryonic and endometrial factors may cause implantation failure [26]. Although technological advances are being rapidly reported, the rates of successful pregnancy remain relatively low, mainly due to implantation failure [25, 27]. Embryo implantation is a complex and highly orchestrated biological process involving multiple pathways. Firm adhesion is essential for embryo implantation.

Cadherins are a superfamily of more than 100 transmembrane glycoproteins that were originally identified as cell adhesion molecules [28]. In recent years, research has revealed new functions of cadherin, including coordinated cell movements, cell sorting, boundary formation, and the maintenance of tissue polarity [29, 30]. E-cadherin plays a critical role in the process of blastocyst formation. Lack of the E-cadherin gene in embryos leads to impaired adhesion junction establishment in the trophectoderm and death in the preimplantation period [31]. Intermediate filaments are vital components of the cytoskeleton, in addition to F-actin and microtubules [32]. Previous studies have shown that keratins are the only cytoplasmic intermediate filaments expressed during preimplantation development [33, 34]. In epithelial tissues, keratins regulate signalling, polarity and mechanics [32]. Additionally, keratin knockout resulted in trophoblast fragility, placental bleeding and lethality after implantation [35–37]. The latest research shows that in the mammalian embryo, intermediate filaments assembled by keratins function as asymmetrically inherited fate determinants [14].

In our study, immunofluorescence staining was performed on mouse preimplantation embryos. We found that the expression of KRT18 gradually increases during early mouse embryo development and is abundant on trophoblast cells of blastocysts. By using an embryo trophoblast-specific knockdown model and a mouse embryo adhesion assay, we found that knockdown of KRT18 by lentivirus decreased mouse embryo adhesion and implantation. Cell motility and invasion play an important role in embryo implantation. By using cell migration and invasion assays, we found that the migratory and invasive abilities of HTR8/SVneo cells were inhibited by KRT18 knockdown. Furthermore, a JEG-3 spheroid-endometrial cell attachment model was applied, and we found that treatment of JEG-3 cells with KRT18 siRNA significantly reduced the JEG3 spheroid attachment rate and outgrowth. MST experiments revealed that KRT18 directly bound to E-cadherin. Knockdown of KRT18 caused a significant decrease in E-cadherin expression. Thus, we speculated that knockdown of KRT18 may influence JEG-3 spheroid-endometrial cell attachment and adhesion through downregulation of E-cadherin. Due to the complexity of the biological activity and underlying molecular mechanisms of these proteins, this study should be regarded as preliminary. Further studies may provide a complete understanding of the mechanism by which KRT18 plays a role in embryonic implantation.

In conclusion, KRT18 is highly expressed on trophoblast cells of blastocysts. Knockdown of KRT18 impaired trophoblast migration and invasion. In addition, trophoblast spheroid adhesion and mouse embryo adhesion and implantation were impaired due to failed interactions with E-cadherin. Our study revealed the function of KRT18 in embryo implantation to some extent and identified a potential target to improve embryo implantation rates and further guide clinical practice.

## Materials and methods

### Mouse embryo collection and culture

The studies were conducted under the approval of the ethical committee of Sir Run Run Shaw Hospital Medical Ethics Committee. Super ovulated eight-week-old ICR female mice were caged individually with ICR males after hCG injection to obtain embryos. The presence of a vaginal plug the next morning was used to define E0.5. Immediately thereafter, the female mice were sacrificed through cervical dislocation. Fertilized zygotes were collected from oviducts and cultured in KSOM (M1450; Easycheck, Nanjing China) at 37°C in a 5% CO_2_ atmosphere. Then, embryos at different stages were harvested for immunofluorescence. An adhesion assay was conducted with blastocysts collected from the uteri of female mice on pregnancy day 3.5. Only expanded blastocysts appearing morphologically normal were included in the study.

### Embryo trophoblast-specific knockdown model

An embryo trophoblast-specific knockdown model was established as described previously [20]. Briefly, mouse blastocysts were obtained at E3.5, and those with normal morphology were selected and incubated for 4 h in lentivirus solution. After several washes, blastocysts were transferred onto Ishikawa cell monolayers and cocultured for 48 h at 37°C in a 5% CO2 atmosphere. After rotation of the plates, the floating embryos were removed. The embryo attachment rate was examined by using an inverted microscope. For in vivo experiments, after incubation for 4 h in lentivirus solution and several washes, blastocysts were transferred into the uteri of foster mice. At 72 h post-transfer, mice were sacrificed, and their uteri were dissected to measure implantation sites.

### Mouse embryo adhesion and in vivo implantation assay

A mouse embryo adhesion assay and in vivo implantation were performed as described previously [38]. Briefly, mouse blastocysts were obtained at E3.5, and then, those with normal morphology were selected and incubated for 4 h in lentivirus solution. After several washes, blastocysts were transferred onto Ishikawa cell monolayers and cocultured for 48 h at 37°C in a 5% CO2 atmosphere. After rotation of the plates, the floating embryos were removed. The embryo attachment rate was examined by using an inverted microscope. For in vivo experiments, after incubation for 4 h in lentivirus solution and several washes, 12 expanded blastocysts with normal morphology from each group were transferred to the uterine horn of pseudopregnant ICR female mice. At 72 h post-transfer, mice were sacrificed, and their uteri were dissected to measure implantation sites.

### Cell culture and reagents

NIH/3T3 and 293T cells were cultured in DMEM (CGM101.05; CellMax, Lanzhou, China). JEG3 cells were cultured in EMEM (11700; Cary, Huzhou, China). HTR8/SVneo cells and Ishikawa cells were cultured in RPMI medium (C11875500BT; Thermo Fisher Biochemical, China). Cells were cultured at 37°C in a 5% CO_2_ atmosphere, and all culture media were supplemented with 100 U/ml penicillin and streptomycin (BL505A; Biosharp, Beijing, China) and 10% foetal bovine serum (CellMax, Lanzhou, China).

KRT18 antibody was purchased from Novus Biologicals (NPB2-67370; Littleton, CO, USA). Phalloidin was purchased from Thermo Fisher Scientific (A22284; USA). KRT18 fusion protein (Ag1260) and E-cadherin fusion protein (Ag15085) were purchased from Proteintech (Wuhan, Hubei, P.R.C.).

### siRNA transfection and lentivirus infection

Control siRNA and KRT18 siRNA were obtained from Shanghai GenePharma (Shanghai, China). For transient transfections, Lipofectamine 3000 (Invitrogen) was used according to the manufacturer’s instructions. The KRT18 siRNA sequences used in this experiment were as follows:

- 5′- GGUUCCCGGAUCUCCGUGUTT-3′,
- 5′- GCUGAUGACUUUAGAGUCATT-3′,
- and 5′- GAGCUAGACAAGUACUGGUTT-3′.

KRT18 knockdown and control lentiviruses were obtained from GeneChem (GeneChem, Shanghai, China). Lentivirus infection was conducted according to the manufacturer’s instructions, and the following KRT18 siRNA sequences were used: sense (5'-3')

- 5'-GGAAGUCCAAGGUCUGGAATT-3', 5'-CCUCCAGACCUUGGAGAUUTT-3', and 5'-CCAUGCAAACUGUGCAGAATT-3'; antisense (5'-3')
- 5'-UUCCAGACCUUGGACUUCCTT-3', 5'-AAUCUCCAAGGUCUGGAGGTT-3', and 5'-UUCUGCACAGUUUGCAUGGTT-3'.

### Immunofluorescence staining and confocal microscopy

For embryo and cell immunofluorescence staining, embryos at different stages or cells were fixed in 4% w/v paraformaldehyde (PFA) for 30 min at room temperature. After, permeabilization was performed with PBS containing 0.5% Triton X-100 for 20 min followed by blocking in 1% w/v bovine serum albumin for 1 h. Then, primary antibodies were applied at 4 °C overnight. Embryos or cells were then treated with Alexa Fluor 568-conjugated and 488-conjugated secondary antibodies after being washed three times with PBS containing 0.01% Triton X-100 and 0.1% Tween-20 for 1 h at room temperature. Hoechst (33342, MA0126; 1 mg/ml; Meilunbio, Dalian, China) staining was used to visualize cell nuclei. A confocal microscope (Carl Zeiss, Oberkochen, Germany) was utilized for fluorescent confocal analysis.

### RNA extraction and Real-Time Quantitative PCR (qRT‒PCR)

Total RNA was isolated using an RNA-Quick Purification Kit (ES Science, China) according to the manufacturer's protocol. The RNA concentration and quality were determined by using a NanoDrop ND2000. Next, Prime HiScript II qRT SuperMix (Vazyme, Nanjing, China) was applied for reverse transcription. Then, real-time PCR was performed using a real-time PCR system (Life Technologies, USA) and SYBR Green qPCR Mix (Beyotime, China). The following primers were used: KRT18 (human, forwards, 5′-TCGCAAATACTGTGGACAATGC-3′; reverse, 5′- GCAGTCGTGTGATATTGGTGT-3′; mus, forwards, 5′-CAGCCAGCGTCTATGCAGG-3′; reverse, 5′-CCTTCTCGGTCTGGATTCCAC-3′), GATA2 (human, forwards, 5′- CAGCAAGGCTCGTTCCTGTT -3′; reverse, 5′- GGCTTGATGAGTGGTCGGT-3′) and GAPDH (human, forwards, 5′- CGGAGTCAACGGATTTGGTCGTAT -3′; reverse, 5′- AGCCTTCTCCATGGTGGTGAAGAC -3′).

### Western blotting

Cell lysates were first treated with RIPA buffer with phosphatase and protease inhibitors. After separation by electrophoresis, the proteins were transferred to PVDF membranes (Millipore). Primary antibodies were incubated with the membranes at 4°C overnight. After 3 washes in TBST, the membranes were treated with HRP-linked anti-rabbit or anti-mouse IgG secondary antibodies (Cell Signaling Technology, USA). After 3 washes in TBST, the bands were imaged using an infrared imaging system (Licor Odyssey CLx infrared system).

### Wound healing assay

For the cell motility assay, HTR8/SVneo cells (2 × 10^5^ cells) that were treated with siKRT18-3 for 24 h were seeded in six-well plates. When the cells grew to 80–90% confluence, a scratch wound was made by using a 200-µL pipette tip. Images were taken at 0 h and 24 h after wounding. The scratch wound size was assessed by ImageJ software.

### Transwell assay

For the transwell assay, HTR8/SVneo cells were treated with siKRT18-3 for 24 h and starved with serum-free DMEM containing 0.2% BSA overnight. Then, 2 × 10^5^ cells were added to the upper transwell chamber, which was coated with Matrigel. DMEM + 10% FBS was added to the lower chamber. After incubation at 37°C for 24 h, cells that adhered to the lower surface were fixed for 15 min with 4% paraformaldehyde. Then, the fixed cells were stained with 0.005% crystal violet and counted in four fields per filter by ImageJ software.

### JEG3 spheroid coculture assay

For the coculture assay, Ishikawa cells were seeded at 0.6 × 10^6^ cells per well in a 12-well plate 24 h before coculture. JEG3 spheroids were generated by shaking cells (at a density of 3 × 10^5^ cells per well of 6-well plate) at 88 rpm for 16–18 h in culture medium under standard cell culture conditions. After Ishikawa culture medium was used to replace the medium (substitute FBS with 1% BSA), the spheroids were selected and transferred onto Ishikawa monolayers. Cocultures were incubated for 1 h, followed by centrifuging the plate at 145 rpm for 10 min. The attachment rate was determined after removing the spheroids that did not bind. The outgrowth of JEG3 spheroids was measured by ImageJ 48 h after transfer.

Cocultures were incubated for 1 h and the plates were centrifuged at 145 rpm for 10 min. The attachment rate was determined after removing the spheroids that did not bind. The outgrowth of JEG3 spheroids was measured by ImageJ 48 h after transfer. The embryo attachment rate was determined by using an inverted microscope.

### MST

MST was performed according to a protocol reported previously [38, 39]. In brief, according to the standard protocol, a RED-NHS protein labelling kit (NanoTemper Technologies, Munich, Germany) was used to label the His-E-cadherin fusion protein. Then, the labelled His-E-cadherin fusion protein was incubated with 2-fold serial dilutions of the GST-KRT18 fusion protein in MST-optimized buffer at a constant concentration (1 µM). Binding reactions were mixed and then incubated at room temperature for 15 min. Then, the mixtures were loaded into the instrument (Monolith NT.115; NanoTemper) after being enclosed in dedicated glass capillaries. The measurement times were as follows: fluorescence before 5 s, MST on 30 s, fluorescence after 5 s, and delay for 25 s. Then, 20 and 40% MST power were used to perform the measurement. Fnorm (normalized fluorescence) = F1 (fluorescence after thermodiffusion)/F0 (initial fluorescence or fluorescence after T-jump). The NanoTemper analysis tool was used to determine the Kd values.

### Statistical analyses

Statistical analyses were performed using GraphPad Prism 8 software (La Jolla, CA, USA). The statistical significance of the differences was analysed by Student’s t test. The mean ± SD was plotted. All statistical tests used a *p* value less than 0.05 to determine statistical significance.

## Data Availability

The data generated in this study are available upon request from the corresponding author.
